# Relative performance of gene- and pathway-level methods as secondary analyses for genome-wide association studies

**DOI:** 10.1186/s12863-015-0191-2

**Published:** 2015-04-08

**Authors:** Genevieve L Wojcik, WH Linda Kao, Priya Duggal

**Affiliations:** Department of Epidemiology, Johns Hopkins University Bloomberg School of Public Health, Baltimore, MD USA; Department of Genetics, Stanford University School of Medicine, Stanford, CA USA

**Keywords:** Genome-wide Association Studies, Gene Set, Biological Pathways

## Abstract

**Background:**

Despite the success of genome-wide association studies (GWAS), there still remains “missing heritability” for many traits. One contributing factor may be the result of examining one marker at a time as opposed to a group of markers that are biologically meaningful in aggregate. To address this problem, a variety of gene- and pathway-level methods have been developed to identify putative biologically relevant associations. A simulation was conducted to systematically assess the performance of these methods. Using genetic data from 4,500 individuals in the Wellcome Trust Case Control Consortium (WTCCC), case–control status was simulated based on an additive polygenic model. We evaluated gene-level methods based on their sensitivity, specificity, and proportion of false positives. Pathway-level methods were evaluated on the relationship between proportion of causal genes within the pathway and the strength of association.

**Results:**

The gene-level methods had low sensitivity (20-63%), high specificity (89-100%), and low proportion of false positives (0.1-6%). The gene-level program VEGAS using only the top 10% of associated single nucleotide polymorphisms (SNPs) within the gene had the highest sensitivity (28.6%) with less than 1% false positives. The performance of the pathway-level methods depended on their reliance upon asymptotic distributions or if significance was estimated in a competitive manner. The pathway-level programs GenGen, GSA-SNP and MAGENTA had the best performance while accounting for potential confounders.

**Conclusions:**

Novel genes and pathways can be identified using the gene and pathway-level methods. These methods may provide valuable insight into the “missing heritability” of traits and provide biological interpretations to GWAS findings.

**Electronic supplementary material:**

The online version of this article (doi:10.1186/s12863-015-0191-2) contains supplementary material, which is available to authorized users.

## Background

In less than one decade after their advent, genome-wide association studies (GWAS) have been remarkably successful and have elucidated many loci for diverse phenotypes [1]. However, there remains “missing heritability”, or the discrepancy between the low amounts of within-population phenotypic variation explained by GWAS results and the higher estimates of narrow-sense heritability [2]. One explanation for this missing heritability is current studies are underpowered to identify contributing genetic variants. The conservative adjustment of the significance threshold (α) for the 1–2.5 million tests results in a p-value significance threshold of 5×10^−7^ [3], and biologically-relevant genetic associations may lie below this threshold, but are ignored in many traditional GWAS.

To improve power within a biologic context, a multitude of gene- and pathway-level methods have been developed for the secondary analyses of GWAS results. These methods aggregate markers into biologically relevant units, such as a gene or pathway, and test the associations within that unit. These methods increase power by combining multiple weak or moderate signals and allow for allelic or locus heterogeneity. An additional motivation for gene- or pathway-level methods is the potential for biologically relevant interpretation as the genes or pathways can be selected based on prior knowledge, or in a genome-wide manner. In comparing these programs, many of the issues surrounding these analytical methods are similar, however the underlying hypotheses and limitations may be distinct.

Gene-level methods look for the joint association of independent signals within a gene. The framework posits that genes contain multiple alleles that may be associated with the outcome of interest, known as allelic heterogeneity, which may only be detected through an aggregate single nucleotide polymorphism (SNP) test. Gene-level methods can be loosely categorized into three groups: classical, updated classical, and novel methods. Classical methods, not specifically developed for genetic data, assume that independent statistics are combined. Updated classical methods use these classical frameworks while accounting for linkage disequilibrium between SNPs within the gene by reducing the dimensions to an effective number of independent SNPs. Novel methods directly estimate the linkage disequilibrium in the genetic data and apply these correlation matrices to statistical estimation. An ideal gene-level method would have high sensitivity and specificity with a low number of false positives. It should also be able to distinguish between multiple independent signals and multiple associations due to linkage disequilibrium.

A pathway, or gene set, is a related collection of genes that can be grouped together based on their biological functions or previous knowledge of disease pathogenesis. The goal of pathway-level methods is to determine if the genetic associations from a GWAS are enriched within a set of genes in a pathway. Most of these pathway methods ignore multiple association signals due to allelic heterogeneity and can be loosely categorized into two groups: competitive and self-contained [4]. Competitive methods assess if strong associations cluster within the gene set at a higher proportion compared to associations outside of the gene set. They depend on the overall distribution of the statistics for all genes genome-wide. Therefore, competitive methods are not ideal for candidate gene studies. Self-contained methods estimate the joint association of the genes within a gene set and typically assume an asymptotic distribution to assess significance, allowing a candidate gene set analysis, but this may be the incorrect distribution for the data.

With a wide variety of published methods, the field still lacks a consensus as to the best practice [4,5]. To address this knowledge gap, we evaluated 21 different methods with readily available software through phenotypic simulation using real genotypic data of 4,500 individuals from the Wellcome Trust Case Control Consortium. We systematically evaluated the relative performance of gene- and pathway-level methods for a case–control GWAS through a simulation of over 17,000 genes and 20 pathways from the Gene Ontology Biological Processes.

## Results

### Gene-level analyses

A total of 11 methods were evaluated: Fisher’s Combination Test (FCT), Sidak’s Combination Test, Simes’ Test, False Discovery Rate (FDR), Truncated Product Method (TPM), GATES (weighted and unweighted), HYST (weighted and unweighted), and VEGAS (using all SNPs and only the top 10% of SNPs per gene). All gene-level methods were able to detect genes with and without a genome-wide statistically significant SNP (*P* < 5×10^−7^). For example, the gene-level program VEGAS using only the top 10% of associated SNPs identified 14 ‘true positive’ genes with *P* < 0.001. Of these 14 genes, only 5 had a SNP with genome-wide significance at *P* < 5×10^−7^.

Of the 11 methods evaluated, Truncated Product Method (TPM), an updated classical method, had the highest sensitivity (63%) (Table 1). However, it also had the second highest proportion of false positives (4.9%) and the second lowest specificity (92.9%). Fisher’s Combination Test, the classical method, had similar results with sensitivity of 59%, specificity of 88.6%, and a proportion of false positives of 5.9%. Sidak’s Combination Test, another classical method, had the lowest sensitivity (18.4%), and the lowest proportion of false positives (0.11%). Newer methods all performed similarly. GATES and HYST, updated classical methods, were nearly identical in their predictions with sensitivity of 24.49%, specificity of 98%, and false positive proportions of 0.17% and 0.16%, respectively. VEGAS, a novel method, had a similar performance with sensitivity of 20.41% and 100% specificity. The proportion of false positives was low at 0.16%. With the exception of Fisher’s Test, Simes’ Test, and TPM, all methods had less than 1% false positives.

**Table 1:** **Performance of gene-level methods**

**Group**	**Method**	**Sensitivity (%)**	**Specificity (%)**	**False positives (%)**	**False negatives (%)**
*Classical*	Fisher	59.18	88.64	5.89	40.82
	Sidak	18.37	97.73	0.11	81.63
	Simes	46.94	97.73	1.33	53.06
	FDR	24.49	97.73	0.13	75.51
*Updated Classical*	TPM	63.04	92.86	4.93	36.96
	GATES	24.49	98.00	0.17	75.51
	WGATES	26.53	98.00	0.16	73.47
	HYST	24.49	98.00	0.16	75.51
	WHYST	24.49	98.00	0.16	75.51
*Novel*	VEGAS	20.41	100.0	0.16	79.59
	VEGAS [10%]	28.57	98.00	0.40	71.43

#### Agreement between programs

Pearson’s correlations were calculated to assess the pairwise *P* -value agreement for the 11 gene-level methods across all 17,000 genes (range 33-98%) (Additional file 1: Figure S5). The highest correlations were found within the previously assigned groups (Table 1); the updated classical methods had high correlation with each other (>95%) with the exception of TPM; the novel methods, the two VEGAS methods (all and top 10%), had similarly high correlation in their *P*-values (88%). Surprisingly, the lowest correlation was between the GATES-associated methods and Simes’ Test (31-34%), considering that GATES is an extended Simes procedure.

#### Stratified results

To examine the influence of effect size on the different methods’ performances, sensitivity was estimated separately for genes simulated to have a large effect size (OR = 2) and genes with a smaller effect size (OR = 1.2) (Table 2). As expected, sensitivity was higher when the effect size was large compared to a smaller effect size, with the exception of Sidak’s Combination Test. This is likely due to Sidak’s test depending upon the minimum P-value within the gene. The sensitivity for the larger effect sizes (OR = 2) was also higher than the overall sensitivity from Table 1. This is consistent with the original simulation framework, as the ‘true positive’ genes that were simulated to have a larger effect size will have smaller p-values on the SNP-level due to increased power, which then translates to the gene-level analyses.

**Table 2:** **Stratified sensitivities by effect sizes and number of causal SNPs under simulation**

**Group**	**Method**	Sensitivity (OR* = 2)	Sensitivity (OR* = 1.2)	**Sensitivity (1 SNP)**	**Sensitivity (2 SNPs)**	**Sensitivity (5 SNPs)**
*Classical*	Fisher	66%	17%	50%	64%	60%
	Sidak	18%	33%	12%	18%	20%
	Simes	50%	17%	50%	50%	45%
	FDR	27%	17%	25%	27%	25%
*Updated Classical*	TPM	68%	20%	57%	63%	65%
	GATES	25%	17%	12%	18%	35%
	GATES [Weighted]	27%	17%	25%	18%	30%
	HYST	25%	17%	12%	18%	40%
	Weighted GATES/HYST	25%	17%	12%	18%	35%
*Novel*	VEGAS	23%	17%	0%	27%	25%
	VEGAS [10%]	32%	17%	0%	32%	40%

Sensitivity and specificity calculated using subset of 49 true positive and 50 true negative genes. False positive and false negative percentages calculated using entire dataset of ~17,000 genes.

*OR = Odds Ratio.

Genes were also stratified based on the number of causal SNPs determined from the simulation (Table 2). Of the 50 true positive genes, 8 genes were simulated using 1 causal SNP, 22 had 2 causal SNPs, and 20 had 5 causal SNPs. Within the classical methods, the sensitivity estimates remained relatively consistent across the causal SNP categories, whereas for the newer methods, sensitivity increased with the number of causal SNPs. This is consistent with their methodology, derived to combine independent signals for a stronger joint association. Neither version of the program VEGAS found genes with only one causal SNP as significant. Within genes with five causal SNPs, VEGAS’s sensitivity increased to 40% from the original overall 28.57%.

### Pathway-level analyses

A total of 10 pathway-level programs were evaluated: ALIGATOR, GenGen, GSA-SNP, GSEA-SNP, MAGENTA, Modified Generalized Fisher Method (MGFM), SNP Ratio Test (SRT), GRASS, HYST, and Plink Set Test (PST). Only the 20 pathways that were simulated to be associated were evaluated (Additional file 1: Table S3). The method with the most significant P-values was HYST, with five pathways having a *P* < 0.001. Pathways in which there were no causal genes (all smaller pathways) did not have significant results by any method. Similarly, no pathways were significant that had less than 12% causal genes. Pathway-level methods can be separated into two groups: competitive (ALIGATOR, GenGen, GSA-SNP, GSEA-SNP, MAGENTA, MGFM, SNP Ratio Test) and self-contained (GRASS, HYST, Plink Set Test). Self-contained tests had more ‘significant’ (*P* < 0.001) findings than the competitive methods. Within the competitive methods, only two pathways were significant and only by GSA-SNP. However, within the five pathways with the most causal genes (12-28%), at least one self-contained method found each significant.

#### Performance of methods

Many of the methods are competitive, with individual pathway’s results depending on the distribution of all evaluated genes. Because of this, the rankings of a pathway may be more informative than the statistical significance. Within each method the *P*-values for the sets were ranked from smallest/strongest (1) to largest/weakest (10). For each pathway, the mean ranking was calculated across the 10 methods for only the larger pathways. Overall, the larger proportions of causal genes were correlated with the higher rankings (correlation of −0.75) (Additional file 1: Figure S6). Correlations between the individual methods’ rankings and the proportion of associated genes ranged from −0.26 (Plink Set Test) to −0.64 (GenGen) (Table 3).

**Table 3:** **Correlation for pathway-level results between rankings within each method and the proportion of associated genes within the pathway using only the 10 larger pathways evaluated, as well as correlation with mean ranking across all programs**

**Group**	**Method**	**Citation**	**Input**	**Correlation with causal proportion**	**Correlation with mean ranking**
				**(95% CI)**	**(95% CI)**
*Competitive*	ALIGATOR	[24]	SNP P-values	−0.6 (−0.89, 0.05)	0.75 (0.22, 0.94)
	GenGen	[25]	SNP P-values	−0.64 (−0.91, −0.02)	0.82 (0.41, 0.96)
	GSA-SNP	[21]	SNP P-values	−0.59 (−0.89, 0.06)	0.78 (0.28, 0.94)
	GSEA-SNP	[22]	Raw Genotypes	−0.6 (−0.89, 0.04)	0.73 (0.18, 0.93)
	MAGENTA	[20]	SNP P-values	−0.63 (−0.9, 0)	0.9 (0.62, 0.98)
	MGFM	[26]	SNP P-values	−0.53 (−0.87, 0.15)	0.7 (0.13, 0.92)
	SRT	[27]	Raw Genotypes	−0.43 (−0.84, 0.27)	0.55 (−0.12, 0.88)
*Self-Contained*	GRASS	[23]	Raw Genotypes	−0.49 (−0.86, 0.2)	0.53 (−0.15, 0.87)
	HYST	[18]	SNP P-values	−0.57 (−0.88, 0.09)	0.84 (0.46, 0.96)
	PST	[11]	Raw Genotypes	−0.26 (−0.76, 0.44)	0.55 (−0.12, 0.88)

#### Correlation between methods

**Figure 1:**
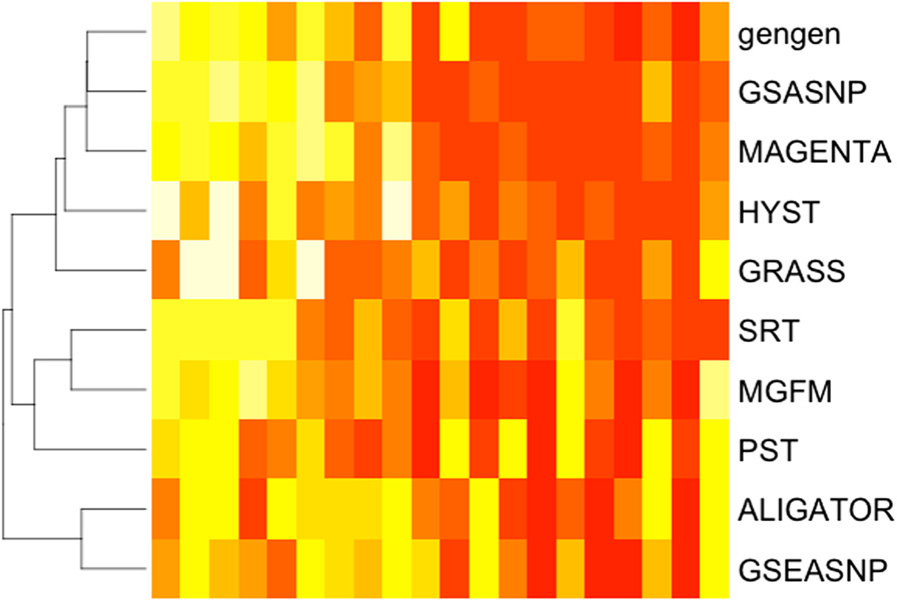
**Heatmap of results for pathway-level methods by the proportion of associated genes within the gene sets.** The results are *P*-values for all pathways using the methods for a complete assessment of performance. Pathways with similar performances will cluster together along the y-axis, as indicated by the dendrogram. Proportion of associated genes (at least one SNP with P < 0.01) is indicated along the x-axis from left (0%) to right (33%). Intensity of color refers to stronger signals (lower *P*-values), which increases with the proportion of associated genes for most methods.

The correlation in P-values between the methods varied from 0.07 (SRT and GRASS) to 0.81 (MAGENTA and GSA-SNP). The SNP Ratio Test (SRT) had the lowest correlations with all the methods. The correlations between a method’s ranking of pathways with the mean ranking for that pathway across all methods varied, with the strongest being MAGENTA (0.9). In a heatmap of the results from the larger pathways, organized from the gene sets with no associated genes to 33% of the genes being associated on the right, three methods cluster together based on their gene set rankings: GenGen, GSA-SNP, and MAGENTA. (Figure 1) They exhibit a trend of weaker P-values and higher rankings with the smaller proportion-associated pathway, and stronger signals in the pathways with more genes associated with outcome (Additional file 1: Table S4).

## Discussion

The goal of gene- and pathway-level methods is to assess enrichment of signals within genes and pathways that might otherwise have been underpowered in a traditional GWAS. The ideal method should be able to detect genes and pathways with small to moderate effect size SNP associations while emphasizing multiple independent signals as opposed to multiple dependent SNPs in linkage disequilibrium. It should have high sensitivity and specificity with a low proportion of false positives. To determine the best method, the relative performance of 11 gene-level and 10 pathway-level methods for GWAS was evaluated through a simulation for 20 different gene sets from Gene Ontology (GO) Biological Processes and over 17,000 genes.

All gene-level methods identified loci that would have otherwise been ignored by a traditional GWAS. The highest sensitivity, or proportion of ‘true positive’ genes that the method determined as associated, was found using Truncated Product Method (63.04%), but this method also had the second lowest specificity (92.86%) and the second highest proportion of false positives (4.93%). This is expected, as the original’s Fisher’s Combination Test (FCT) is prone to test statistic inflation because it combines *P*-values incorrectly assumed to be independent, as linkage disequilibrium between genic SNPs creates correlation structure. The Truncated Product Method (TPM) is an adaptation of FCT, only considering P-values under a certain threshold (0.1 in this case) and combining them in a similar manner. This generalized inflation leads to the highest sensitivity, paired with the second highest proportion of false positives next to FCT. The highest specificity was found with VEGAS, a more conservative approach with a sensitivity of 20.41%. VEGAS adjusts for linkage disequilibrium directly by estimating the correlation structure with HapMap data, or the raw genotype data from the GWAS, and integrating it into the statistics. This may be a conservative procedure, as VEGAS also has the highest level of false negatives among methods with similar false positive proportions, especially when it comes to smaller effect sizes. An additional option is to use VEGAS with only the top 10% of SNPs within a gene, resulting in higher sensitivity (29%) while maintaining high specificity (98%) and a low proportion of false positives (0.40%).

Analyses stratified by the simulated effect sizes or the number of causal SNPs reinforces the framework underlying genome-wide association studies assuming a polygenic model. Smaller effect sizes are underrepresented in SNPs with *P* < 0.01. The original 226 genes were divided evenly between the two effect sizes (OR = 1.2 vs OR = 2.0) within the simulation. However, only 6 of the 49 true positive genes had the smaller effect size (OR = 1.2). This is consistent with larger effect sizes having increased power compared to smaller effect sizes within the GWAS model [6]. Because true positive genes required at least one SNP with *P* < 0.01, the underpowered smaller effect sizes were not represented well in this group. Sensitivity was increased for all methods within the stronger effect genes. The number of independent causal SNPs also had a large effect on the method’s sensitivity. For most methods, sensitivity increased with the number of causal SNPs or independent signals. VEGAS, using either all of the SNPs within the gene or just the top 10% of associated SNPs, did not detect genes which had only one causal SNP while sensitivity was increased within genes with 2 or 5 independent causal SNPs. If the underlying hypothesis is that there are multiple causal SNPs within a gene that could be contributing to the outcome, as is the case with allelic heterogeneity, then VEGAS will help to differentiate between genes that have multiple signals due to linkage disequilibrium or multiple independent signals.

All methods had a small amount of bias in regards to physical gene size, with the absolute number of SNPs in the gene having more of an effect (Additional file 1: Figure S9). Consistent with violating the underlying assumption of independence between association signals within FCT, an increase in the number of SNPs resulted in a less accurate analysis. The proportion of causal SNPs to the total number of SNPs in the gene influenced the accuracy of VEGAS using the top 10% SNPs, increasing the accuracy with the higher proportion of causal SNPs. This is consistent with the aim of gene-level methods to elucidate genes with multiple independent signals that would otherwise be ignored in a traditional GWAS.

When choosing a gene-level method for the secondary analysis of GWAS, it is important to take into consideration how the results will be used. If the goal of the investigator is to generate an all-inclusive list for low cost follow-up, the sensitivity should be maximized with less regard to the specificity or proportion of false positives, such as with the Truncated Product Method. If instead the goal of the investigator is to follow-up with a high-cost experiment, it may be more important to minimize false positives with Sidak’s Combination Test. However, for the average investigator seeking to elucidate loci that are below a genome-wide significance threshold but biologically relevant, it is likely that a balance of sensitivity and specificity will be most useful. Of the gene-level methods evaluated, VEGAS using only the top 10% of SNPs within the gene region offers high sensitivity (28.6%) with less than 1% false positives, while being able to distinguish between multiple independent causal loci and multiple signals due to linkage disequilibrium.

For the pathway-level programs, the underlying hypothesis for these methods is that multiple genes will be associated with the phenotype, a true polygenic model, and that these associated genes will be clustered in sets of genes that have a biological relationship with one another. As hypothesized, these pathway methods found enriched gene sets with a higher proportion of associated genes as compared to gene sets with a lower proportion of associated genes. The methods that ignored genic architecture and collapsed all SNPs within the genes into a single pathway unit (SRT, PST) had the lowest correlations with the proportion of causal genes. These methods test for the joint association of SNPs within the gene set and not necessarily the enrichment of associated genes within a gene set. However, these methods and the Modified Generalized Fisher’s Method (MFGM) are the only methods suited to handle allelic heterogeneity. Other methods assign the gene-level *P*-value as the minimum SNP *P*-value found in the genic region, ignoring the relevance of additional independent signals within this region.

Three methods clustered together based on their results (GSA-SNP, GenGen, and MAGENTA), showing high correlation between the proportion of causal genes and the ranking of gene sets. As they are all competitive methods that do not depend upon a pre-defined distribution, but rather the relative enrichment of the gene set compared to all other genes evaluated, the rankings may be more important than the absolute P-value. It is important to note that when interpreting results, users should not disregard results strictly based on a significance threshold but also examine rankings.

There are limitations with this analysis. The list of programs evaluated is not exhaustive as it was curated to reflect methods with publically available software designed explicitly for GWAS. Therefore, it does not include computationally intensive methods that would be more appropriate for a smaller number of candidate genes or gene sets, such as Gamma Method (GM) approaches [7] for self-contained gene sets and other principal components-based approaches [8] for genes. The evaluated methods were all scalable to genome-wide datasets, provided the researcher has access to high-performance computing resources. An additional limitation inherent in all simulation studies is that the results are dependent upon the model and its assumptions. Additional repeated simulations were conducted to assess the stability of the simulation model, as well as the influence of significance thresholds. Estimates were found to be stable across different simulations (Additional file 1: Figures S7 and S8) and the relative performance of methods was consistent using a range of significance thresholds (Additional file 1: Tables S6–S8). Another possible limitation is that the simulation model assumes SNP associations will be independent from one another and will follow a polygenic additive model. While this is simplistic, an additive model is commonly assumed when evaluating SNP associations in case–control GWAS through regression. The gene-level methods’ results do not depend on the overall distribution of associations, therefore the extent of polygenicity is irrelevant. On the other hand, the presence of polygenicity is vital to the use of pathway-level methods, which seeks numerous associated genes within a pathway. In short, although the model is simplistic and may not be entirely reflective of the true pathogenesis of some complex traits, it is valid and should not influence the relative performance of both gene- and pathway level analyses for GWAS.

It is also important to keep in mind the respective limitations of the analytical methods themselves. Gene-level methods seek to aggregate independent signals within a gene. Their utility will depend upon the underlying genetic architecture of specific diseases. If there is only one causal SNP within the gene, these methods will not have increased power compared to a traditional GWAS. On the other hand, if the hypothesis is that there are numerous independent moderate effect risk loci within a gene, these methods will be able to aggregate them for statistical enrichment. Pathway-level methods for GWAS do not evaluate gene-gene interactions or pinpoint the downstream effects of polymorphisms in a gene. Instead, these methods offer a visualization of the data that did not reach genome-wide significance but may be suggestive and biologically relevant to the phenotype of interest. By determining which pathways are enriched for signal within a GWAS, candidate genes and regions are highlighted and may identify relationships between seemingly disparate phenotypes that have a similar pathogenesis.

## Conclusions

Gene- and pathway-level methods for genome-wide association studies remain useful tools for conceptualizing GWAS results beyond the traditional SNP-level results that require a strict significance threshold. Gene-level methods will help elucidate multiple independent statistical signals in an easily interpretable manner by highlighting specific genes. By examining the relative importance of different gene sets with the results, pathway-level methods may generate hypotheses for biological processes involved in the phenotype of interest. Both classes of methods offer researchers a more complete understanding of their genome-wide association study within a biological context.

## Methods

### Genotypic data

For the simulation we used the common controls from the Wellcome Trust Case–control Consortium 2 (WTCCC2), as per the WTCCC2 Data Access Agreement. Data from the 1958 Birth Cohort (N = 2,930) and the National Blood Service (N = 2,737) were previously genotyped using a custom Illumina 1.2 M SNP array [9]. Standard quality control measures were used: genotyping missingness <5%, individual missingness <5%, minor allele frequency (MAF) > 1%, Hardy-Weinberg equilibrium P-value > 10^−5^. Individuals were screened for cryptic relatedness and first-degree relatives were removed. The inbreeding coefficient F was estimated and individuals more than 5 standard deviations away from the mean were removed. Principal components analysis (PCA) was conducted to ensure a homogenous sample without outliers using EIGENSTRAT [10]. PCA was conducted using a subset of markers that were selected to be independent (maximum r^2^ cutoff of 0.05) using the program Plink [11]. Regions known to be ancestry-informative were removed (e.g. *lactase, MHC*) for PCA. After employing quality control measures, the final data set consisted of a total of 4,500 individuals and 906,298 SNPs.

### Gene and pathway selection

Pathways were downloaded from the Molecular Signature Database (MSigDB) for the Gene Ontology Biological Processes [12]. There were 825 processes identified and from these a subset of 20 “pathways” were selected: 10 with greater than the median size of 28 genes and 10 with less than the median. From each selected pathway, a subset of genes were categorized as causal. Within each group: 4 pathways had only 1 causal gene, 4 pathways had 20% of their genes designated causal and 2 pathways had 50% causal genes. Genes were removed from the causal gene list if they were in numerous pathways. The number of causal SNPs and the effect size was varied by gene. Causal SNPs were selected by identifying independent SNPs (maximum pairwise r^2^ of 0.2) within the genic region and the 20 kilobase (kb) flanking regions using the program Tagger [13]. From these independent SNPs in these gene regions, a subset of 1, 2, or 5 causal SNPs were selected. A 20 kb flanking region was used to define the gene region based on prior evidence that only 5% of eQTLs lie further than 20 kb away from the transcription start site (TSS) [14]. All SNPs within a gene were assigned the same effect size: an odds ratio (OR) of 1.2 (small) or 2.0 (larger). This resulted in 602 causal SNPs from 226 genes in 20 pathways (Additional file 1: Figure S1).

### Phenotype simulation

The genotypes for the 602 causal SNPs were converted to an additive format by the number of minor alleles per person. The allele dosage was then multiplied by the log-transformed odds ratio assigned to a particular gene to be consistent with logistic regression assuming an additive model. Genotypic scores were summed across all locations per individual to generate a liability score, which was then standardized. This liability score represented the additive effects from all causal SNPs. From these liability scores an individual was assigned case/control status using a binomial distribution (Additional file 1: Figure S2). The simulation was designed to have an equal number of cases and controls (n = 2,250).

### Genome-wide association analysis

The test of association was performed for an additive model using an unadjusted logistic regression in Plink [11]. The genome-wide threshold for significance was a P-value < 5×10^−7^ (Additional file 1: Figures S3 and S4). To evaluate the performance of methods in a smaller sample size (n = 500), a random subset of individuals was selected and analyzed. Additionally, we evaluated the efficiency of the model by simulating the phenotype 100 times for a subset of 20 “causal” SNPs to create a distribution of simulated effect sizes (Additional file 1: Figure S7). The original simulation was consistent with this distribution.

### Gene-level methods

A total of 11 methods from three categories were evaluated in the gene-level simulation. For the Classical Methods we evaluated the Fisher’s Combination Test (FCT), Sidak’s Combination Test (SCT), Simes’ Test (ST) and the False Discovery Rate (FDR) Correction [15]. For the Updated Classical Methods we evaluated a Truncated Product Method (TPM) [16], as well as the GATES (weighted and unweighted) and HYST (weighted and unweighted) methods [17,18]. For the Novel methods we evaluated VEGAS using all SNPs and using only the top 10% of associated SNPs [19]. Detailed descriptions of these methods are in the Additional file 1.

### Pathway-level methods

We evaluated 10 pathway-level methods: Meta-Analysis Gene-set Enrichment of variaNT Analysis (MAGENTA) [20], Plink Set Test [11], Gene Set Analysis for SNPs (GSA-SNP) [21], Gene Set Enrichment Analysis for SNP data (GSEA-SNP) [22], Gene Set Ridge Regression in Association Studies (GRASS) [23], Association List Go AnnoTatOr (ALIGATOR) [24], GenGen [25], Hybrid Set-Based Test for Genome-wide Association Studies (HYST) [18], a Modified Generalized Fisher’s Method (MGFM) [26], and SNP Ratio Test (SRT) [27]. Detailed descriptions of these methods are in the Additional file 1. Methods were divided into two categories: competitive (ALIGATOR, MAGENTA, GSA-SNP, GSEA-SNP, GenGen, MGFM, and SRT) and self-contained (GRASS, HYST, PST). All methods allow the user to define the assignment of SNPs to genes, which were assigned to the translated region and 20 kb flanking regions.

### Evaluation

#### Gene

For gene-level analyses, a p-value threshold of 0.001 was used to determine statistical significance for all analyses. True positive genes were genes on the original causal gene list within the simulation, and had at least one SNP with a P-value < 0.01 to ensure that true positive genes had signal on a SNP-level. Due to the stochastic element of the simulation, not all genes contributed equally to the liability score. The true negative genes were those not within 50 kb of any causal genes. This resulted in 49 true positive and over 17,000 true negative genes that were used to measure the proportion of false negatives and false positive results. This differs from a type I error (false positive) rate because only one simulation was conducted, preventing repeated testing of the same null hypothesis. Sensitivity and specificity were measured using the 49 true positive genes and a randomly selected subset of 50 true negative genes to prevent inflation of cell size. Sensitivity was calculated as the proportion of “true positive” genes with *P* < 0.001. Specificity was calculated as the proportion of the “true negative” genes with *P* > 0.001. A number of thresholds were used to calculate sensitivity, specificity, and proportion of false positives, ranging from a baseline of 0.001 to a stringent Bonferroni correction of 0.05/17,000 (2.9E-0.6). The relative performance of methods remained consistent across different *P*-value thresholds. (Additional file 1: Tables S6–S8). For a subset of gene-level programs (VEGAS, Fisher’s Combination Test), the entire simulation was conducted 10 times to assess the stability of the simulation. The proportion of false positives and the specificity were found to be extremely stable (Additional file 1: Figure S8). To address potential biases, sensitivity was recalculated with genes stratified by their simulated effect sizes or by the number of causal SNPs within a gene. The effect of gene size, SNP density, the proportion of causal SNPs to all SNPs in a gene, the number of causal SNPs, and the proportion of causal SNPs to the physical gene size were all evaluated regressing the accuracy of results with being true negatives or positives on these factors.

#### Pathway

For the pathway-level analyses, there were a small number of evaluated pathways with causal genes. While pathways were simulated to have a certain percentage of causal genes, the true causal genes were genes within the pathways that had at least one SNP with a *P*-value <0.01. Therefore, 5 out of the 20 pathways had no causal genes and are annotated as such (Additional file 1: Table S3). A qualitative analysis was conducted examining the relationship between % causal genes and statistical significance as evaluated by the P-values from the analysis. Because many of the methods are competitive, the relationship between the percentage of causal genes and the rankings of the pathways was evaluated. Only the 10 larger pathways were used for the estimation of correlation with the percentage of causal genes to avoid an overrepresentation of pathways without any causal genes (null gene sets). All correlations were estimated using Pearson’s correlation. While only the results for a subset of the pathways are presented, the entire MSigDB Gene Ontology Biological Processes set was evaluated for all competitive methods.

### Sensitivity to model selection

The simulation schematic assumes a normally distributed underlying liability score within the general population. By sampling 1:1 cases and controls, it assumes a 50% phenotypic prevalence. Because this may not be realistic for many GWAS, additional phenotypic simulations were conducted to compare the relative performance of a population with 14% prevalence (fewer cases than controls) both in a case-cohort (633 cases compared to 3,867 controls) as well as case–control (633 cases, 633 controls) study design. Fisher’s combination test (FCT) and VEGAS using the top 10% of SNPs were used to evaluate the data for consistency. Relative performance was found to be similar to the original analysis with 50% prevalence (Additional file 1: Table S5).

## Additional file

Additional file 1:
**Supplementary Tables and Figures.**

## Abbreviations

SNP: Single Nucleotide Polymorphism
GWAS: Genome-wide Association Study
