# Supplementary material for: Relative performance of gene- and pathway-level methods as secondary analyses for genome-wide association studies
Source: BMC Genet. 2015 Apr 8;16:34. doi: 10.1186/s12863-015-0191-2 (PMC4391470; doi:10.1186/s12863-015-0191-2)
Supplement: Additional file 1: — Supplementary Tables and Figures. [file 12863_2015_191_MOESM1_ESM.docx]

## Supplementary Tables and Figures

| ***Group*** | ***Method/Program*** | ***Sensitivity*** | ***Specificity*** | ***% False Positive*** | ***% False Negative*** |
| --- | --- | --- | --- | --- | --- |
| *Classical* | Fisher’s | 12.24 | 95.45 | 5.32 | 87.76 |
|  | Sidak’s | 0.00 | 100.00 | 0.03 | 100.00 |
|  | Simes’ | 4.08 | 100.00 | 0.98 | 95.92 |
|  | FDR | 0.00 | 100.00 | 0.05 | 100.00 |
| *Updated* | GATES | 0.00 | 100.00 | 0.10 | 100.00 |
|  | Weighted GATES | 0.00 | 100.00 | 0.13 | 100.00 |
|  | HYST | 0.00 | 100.00 | 0.10 | 100.00 |
|  | Weighted HYST | 0.00 | 100.00 | 0.12 | 100.00 |
| *Novel* | VEGAS | 0.00 | 100.00 | 0.10 | 100.00 |
|  | VEGAS, Top 10% | 0.00 | 100.00 | 0.26 | 100.00 |

***Table S1: Performance of Gene-Level Methods in Smaller Sample Size***

| ***Group*** | ***Method*** | ***Sensitivity*** | ***Specificity*** | ***% False Positive*** | ***% False Negative*** |
| --- | --- | --- | --- | --- | --- |
| *Classical* | Fisher’s | 60.87 | 90.91 | 8.91 | 39.13 |
|  | Sidak’s | 8.70 | 100.00 | 0.54 | 91.30 |
|  | Simes’ | 100.00 | 93.18 | 8.28 | 0.00 |
|  | FDR | 8.70 | 100.00 | 0.75 | 91.30 |
| *Updated* | GATES | 0.00 | 97.73 | 1.09 | 100.00 |
|  | Weighted GATES | 0.00 | 97.73 | 1.11 | 100.00 |
|  | HYST | 0.00 | 97.73 | 1.05 | 100.00 |
|  | Weighted HYST | 4.35 | 97.73 | 1.01 | 95.65 |
| *Novel* | VEGAS | 0.00 | 100.00 | 0.92 | 100.00 |
|  | VEGAS, Top 10% | 30.43 | 100.00 | 2.15 | 69.57 |

***Table S2: Performance of Gene-Level Methods in Smaller Sample Size,*** α=0.01.

| ***Biological Process*** | ***# Genes*** | ***# P<0.01*** | ***% P<0.01*** | ***Competitive Programs*** | | | | | | | ***Self-Contained Programs*** | | |
| --- | --- | --- | --- | --- | --- | --- | --- | --- | --- | --- | --- | --- | --- |
|  |  |  |  | ***ALI*** | ***GenGen*** | ***GSA*** | ***GSEA*** | ***MAG*** | ***MGFM*** | ***SRT*** | ***GRASS*** | ***HYST*** | ***PST*** |
| Lipid Transport | 29 | 8 | 27.59% | 0.244 | 0.005 | *4.14E-04* | 0.186 | 0.022 | 0.073 | 0.02 | *<0.001* | *5.42E-08* | 0.06 |
| Membrane Lipid Metabolic Process | 98 | 15 | 15.31% | 0.198 | 0.143 | 0.056 | 0.057 | 0.018 | 0.039 | 0.127 | 0.014 | 0.02 | *<0.001* |
| Anatomical Structure Morphogenesis | 363 | 50 | 13.77% | 0.457 | 0.055 | *7.94E-06* | 0.496 | 0.161 | 0.026 | 0.549 | *<0.001* | 0.1 | 1 |
| Establishment and/or Maintenance of Chromatin Architecture | 71 | 9 | 12.68% | 0.983 | 0.036 | 0.113 | 0.896 | 0.033 | 0.485 | 0.002 | 0.116 | *7.58E-09* | 0.06 |
| G-Protein Coupled Receptor Protein Signaling Pathway | 332 | 40 | 12.05% | 0.515 | 0.663 | 0.005 | 0.267 | 0.026 | 0.358 | 0.691 | *<0.001* | 0.19 | 0.99 |
| Cellular Defense Response | 55 | 6 | 10.91% | 0.642 | 0.104 | 0.009 | 0.829 | 0.126 | 0.027 | 0.026 | 0.374 | 0.04 | 0.01 |
| Leukocyte Activation | 65 | 7 | 10.77% | 0.996 | 0.761 | 0.534 | 0.955 | 0.944 | 0.047 | 0.246 | 0.146 | 0.74 | 0.45 |
| Response to Hypoxia | 28 | 3 | 10.71% | 0.915 | 0.116 | 0.409 | 0.658 | 0.312 | 0.470 | 0.621 | 0.055 | 0.12 | 0.15 |
| T-Cell Activation | 41 | 4 | 9.76% | 0.929 | 0.475 | 0.275 | 0.823 | 0.903 | 0.533 | 0.241 | 0.089 | 0.24 | 0.25 |
| Regulation of DNA Binding | 44 | 4 | 9.09% | 0.962 | 0.838 | 0.949 | 0.918 | 0.93 | 0.368 | 0.287 | 0.907 | 0.18 | 0.87 |

***Table S3: Results (P-values) from Pathway Analysis for Larger Pathways. P-values are reported as is from the program. P-values annotated as <0.001 are from adaptive permutations, in which the analysis was halted after this threshold was met.***

| ***Group*** | ***Program*** | ***All Pathways*** | | ***Larger Pathways*** | |
| --- | --- | --- | --- | --- | --- |
|  |  | ***Correlation*** | ***Correlation*** | ***Correlation*** | ***Correlation*** |
|  |  | ***(P)*** | ***(-logP)*** | ***(P)*** | ***(-logP)*** |
| *Competitive* | ALIGATOR | -0.2247 | 0.2741 | -0.6555 | 0.6733 |
|  | GenGen | -0.6327 | 0.5557 | -0.5054 | 0.8130 |
|  | GSA-SNP | -0.7334 | 0.5747 | -0.4781 | 0.5347 |
|  | GSEA-SNP | -0.1685 | 0.2671 | -0.6522 | 0.5794 |
|  | MAGENTA | -0.6372 | 0.5994 | -0.5116 | 0.6189 |
|  | MGFM | -0.3561 | 0.3840 | -0.5404 | 0.5989 |
|  | SRT | -0.7438 | 0.5891 | -0.3482 | 0.3612 |
| *Self-Contained* | GRASS | -0.3052 | 0.3196 | -0.4036 | 0.6293 |
|  | HYST | -0.5705 | 0.5580 | -0.3746 | 0.6507 |
|  | PST | -0.1037 | 0.2315 | -0.2939 | 0.2515 |

***Table S4: Correlation for Pathway-Level Results between P-values (and –log_10_ transformed P-values) with Proportion of Associated Genes Using All Pathways, as well as only the Larger Pathways***

| ***Scenario*** | ***Program*** | ***Sensitivity*** | ***Specificity*** | ***False Positives*** |
| --- | --- | --- | --- | --- |
| Prevalence=50%  N=4500 | Fisher’s Combination Test | 59.18% | 88.64% | 5.89% |
|  | VEGAS, Top 10% | 28.57% | 98.00% | 0.40% |
| Prevalence=20%  N=4500 | Fisher’s Combination Test | 63.27% | 100% | 5.97% |
|  | VEGAS, Top 10% | 36.73% | 100% | 0.46% |
| Prevalence=20%  N=1266 | Fisher’s Combination Test | 42.86% | 100% | 5.90% |
|  | VEGAS, Top 10% | 22.45% | 100% | 0.32% |

***Table S5: Performance of FCT and VEGAS (using top 10% of SNPs) under different sampling scenarios***

| **Significance Threshold** | **Fisher’s** | **Sidak’s** | **Simes’** | **FDR** | **TPM** | **GATES** | **HYST** | **WGATES** | **WHYST** | **VEGAS** | **VEGAS,**  **Top 10%** |
| --- | --- | --- | --- | --- | --- | --- | --- | --- | --- | --- | --- |
| *1.00E-03* | 6.1E-02 | 1.1E-03 | 1.4E-02 | 1.4E-03 | 5.0E-02 | 0 | 0 | 1.8E-03 | 1.8E-03 | 1.6E-03 | 4.1E-03 |
| *8.89E-04* | 5.9E-02 | 1.0E-03 | 1.2E-02 | 1.1E-03 | 5.0E-02 | 0 | 0 | 1.8E-03 | 1.7E-03 | 1.4E-03 | 3.8E-03 |
| *7.78E-04* | 5.8E-02 | 9.0E-04 | 1.1E-02 | 1.1E-03 | 4.9E-02 | 0 | 0 | 1.7E-03 | 1.6E-03 | 9.0E-04 | 3.6E-03 |
| *6.68E-04* | 5.7E-02 | 6.0E-04 | 9.5E-03 | 7.0E-04 | 4.8E-02 | 0 | 0 | 1.4E-03 | 1.6E-03 | 6.0E-04 | 2.9E-03 |
| *5.57E-04* | 5.5E-02 | 5.0E-04 | 7.9E-03 | 6.0E-04 | 4.6E-02 | 0 | 0 | 1.2E-03 | 1.4E-03 | 6.0E-04 | 2.4E-03 |
| *4.46E-04* | 5.3E-02 | 4.0E-04 | 6.0E-03 | 4.0E-04 | 4.5E-02 | 0 | 0 | 9.0E-04 | 1.1E-03 | 4.0E-04 | 1.2E-03 |
| *3.35E-04* | 5.1E-02 | 4.0E-04 | 4.7E-03 | 4.0E-04 | 4.3E-02 | 0 | 0 | 9.0E-04 | 9.0E-04 | 4.0E-04 | 8.0E-04 |
| *2.25E-04* | 4.7E-02 | 3.0E-04 | 3.2E-03 | 3.0E-04 | 4.0E-02 | 0 | 0 | 8.0E-04 | 7.0E-04 | 4.0E-04 | 8.0E-04 |
| *1.14E-04* | 4.3E-02 | 3.0E-04 | 1.5E-03 | 3.0E-04 | 3.7E-02 | 0 | 0 | 4.0E-04 | 4.0E-04 | 4.0E-04 | 7.0E-04 |
| *2.94E-06* | 2.6E-02 | 1.0E-04 | 4.0E-04 | 1.0E-04 | 2.2E-02 | 0 | 0 | 2.0E-04 | 1.0E-04 | 3.0E-04 | 3.0E-04 |

***Table S6: Proportion of False Positives for all Gene-Level Programs using difference significance thresholds from 0.001 to a Bonferroni corrected value of 0.05/17,000 (2.9E-6).***

| **Significance Threshold** | **Fisher’s** | **Sidak’s** | **Simes’** | **FDR** | **TPM** | **GATES** | **HYST** | **WGATES** | **WHYST** | **VEGAS** | **VEGAS,**  **Top 10%** |
| --- | --- | --- | --- | --- | --- | --- | --- | --- | --- | --- | --- |
| *1.00E-03* | 0.59 | 0.18 | 0.47 | 0.24 | 0.63 | 0.24 | 0.24 | 0.27 | 0.24 | 0.20 | 0.29 |
| *8.89E-04* | 0.59 | 0.18 | 0.41 | 0.20 | 0.63 | 0.24 | 0.22 | 0.24 | 0.24 | 0.20 | 0.29 |
| *7.78E-04* | 0.59 | 0.18 | 0.37 | 0.20 | 0.61 | 0.22 | 0.22 | 0.24 | 0.24 | 0.20 | 0.29 |
| *6.68E-04* | 0.59 | 0.18 | 0.35 | 0.18 | 0.61 | 0.22 | 0.22 | 0.24 | 0.22 | 0.18 | 0.29 |
| *5.57E-04* | 0.59 | 0.18 | 0.31 | 0.18 | 0.61 | 0.22 | 0.22 | 0.22 | 0.22 | 0.16 | 0.27 |
| *4.46E-04* | 0.59 | 0.18 | 0.31 | 0.18 | 0.59 | 0.22 | 0.22 | 0.22 | 0.22 | 0.16 | 0.22 |
| *3.35E-04* | 0.59 | 0.18 | 0.31 | 0.18 | 0.57 | 0.20 | 0.18 | 0.20 | 0.18 | 0.16 | 0.22 |
| *2.25E-04* | 0.55 | 0.16 | 0.29 | 0.16 | 0.57 | 0.20 | 0.18 | 0.18 | 0.16 | 0.16 | 0.22 |
| *1.14E-04* | 0.51 | 0.12 | 0.24 | 0.12 | 0.54 | 0.18 | 0.16 | 0.16 | 0.16 | 0.14 | 0.16 |
| *2.94E-06* | 0.39 | 0.04 | 0.12 | 0.06 | 0.41 | 0.06 | 0.10 | 0.06 | 0.10 | 0.08 | 0.08 |

***Table S7: Sensitivity of Programs using difference significance thresholds from 0.001 to a Bonferroni corrected value of 0.05/17,000 (2.9E-6).***

| **Significance Threshold** | **Fisher’s** | **Sidak’s** | **Simes’** | **FDR** | **TPM** | **GATES** | **HYST** | **WGATES** | **WHYST** | **VEGAS** | **VEGAS,**  **Top 10%** |
| --- | --- | --- | --- | --- | --- | --- | --- | --- | --- | --- | --- |
| *1.00E-03* | 0.90 | 0.97 | 0.97 | 0.97 | 0.93 | 0.97 | 0.97 | 0.97 | 0.97 | 1.00 | 0.97 |
| *8.89E-04* | 0.90 | 0.97 | 0.97 | 0.97 | 0.93 | 0.97 | 0.97 | 0.97 | 0.97 | 1.00 | 0.97 |
| *7.78E-04* | 0.90 | 0.97 | 0.97 | 0.97 | 0.93 | 0.97 | 0.97 | 0.97 | 0.97 | 1.00 | 0.97 |
| *6.68E-04* | 0.90 | 0.97 | 0.97 | 0.97 | 0.93 | 0.97 | 0.97 | 0.97 | 0.97 | 1.00 | 0.97 |
| *5.57E-04* | 0.90 | 0.97 | 0.97 | 0.97 | 0.93 | 0.97 | 0.97 | 0.97 | 0.97 | 1.00 | 0.97 |
| *4.46E-04* | 0.92 | 0.97 | 0.97 | 0.97 | 0.93 | 0.97 | 0.97 | 0.97 | 0.97 | 1.00 | 1.00 |
| *3.35E-04* | 0.92 | 0.97 | 0.97 | 0.97 | 0.93 | 0.97 | 0.97 | 0.97 | 0.97 | 1.00 | 1.00 |
| *2.25E-04* | 0.92 | 1.00 | 0.97 | 1.00 | 0.93 | 0.97 | 0.97 | 1.00 | 0.97 | 1.00 | 1.00 |
| *1.14E-04* | 0.92 | 1.00 | 0.97 | 1.00 | 0.95 | 1.00 | 0.97 | 1.00 | 0.97 | 1.00 | 1.00 |
| *2.94E-06* | 0.95 | 1.00 | 0.97 | 1.00 | 0.98 | 1.00 | 1.00 | 1.00 | 1.00 | 1.00 | 1.00 |

***Table S8: Specificity of Programs using difference significance thresholds from 0.001 to a Bonferroni corrected value of 0.05/17,000 (2.9E-6).***

| **Method** | **Cut-off** | **False Positive Proportion** | **Sensitivity** | **Specificity** |
| --- | --- | --- | --- | --- |
| *Hsu (2013)* | 0.1 | 0.053 (0.049,0.056) | 0.6 (0.45,0.74) | 0.91 (0.78,0.97) |
|  | 0.2 | 0.062 (0.058,0.065) | 0.6 (0.45,0.74) | 0.86 (0.73,0.95) |
|  | 0.5 | 0.062 (0.058,0.066) | 0.67 (0.52,0.8) | 0.89 (0.75,0.96) |
| *Zaykin (2003)* | 0.1 | 0.049 (0.046,0.053) | 0.63 (0.48,0.77) | 0.93 (0.81,0.99) |
|  | 0.2 | 0.059 (0.055,0.063) | 0.63 (0.48,0.77) | 0.86 (0.72,0.95) |
|  | 0.5 | 0.059 (0.055,0.063) | 0.67 (0.52,0.8) | 0.88 (0.75,0.96) |
| *Fisher (original)* | 1 | 0.059 (0.055,0.063) | 0.59 (0.44,0.73) | 0.89 (0.75,0.96) |

***Table S9:*** *Performance of Fisher’s Combination Test, and adaptations including the Truncated Product Method (TPM) from Zaykin (2002) and the Truncated Product Method using a binomial mixture of gamma distributions described in Hsu (2013).*

***
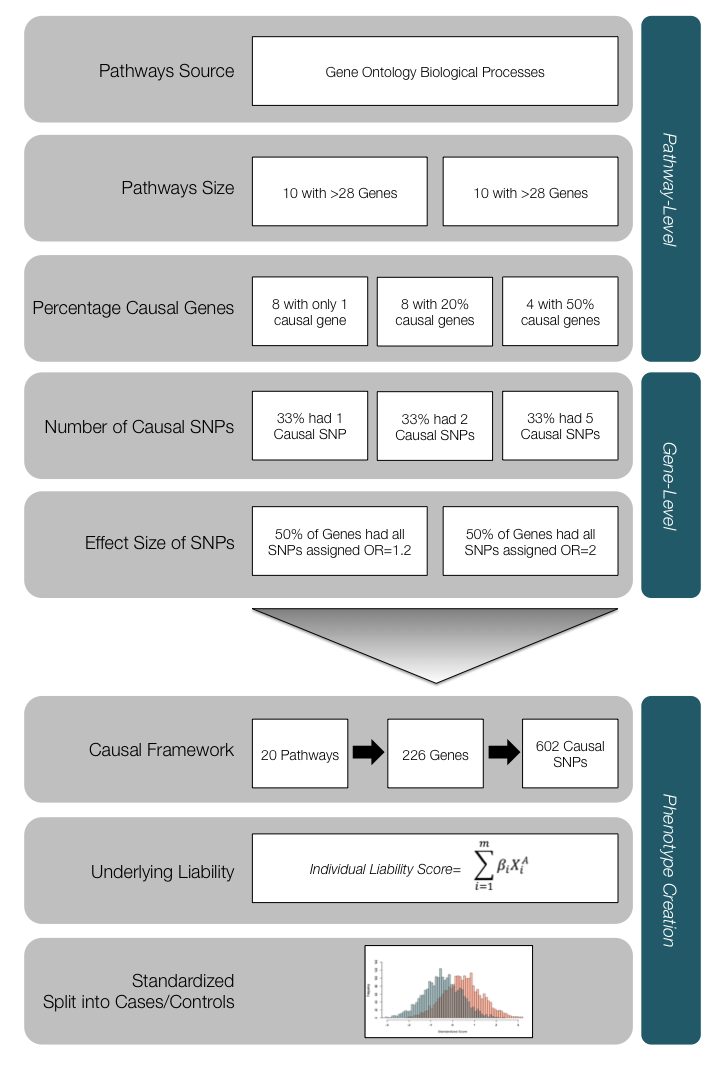
***

***Figure S1: Simulation Schematic***


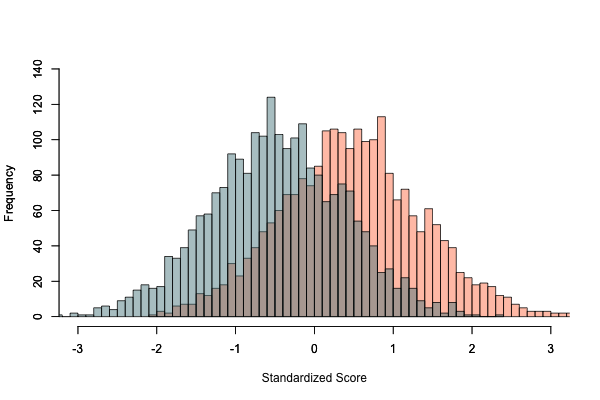


***Figure S2: Frequencies of the standardized liability scores by simulated case (pink) and control (blue) status.***


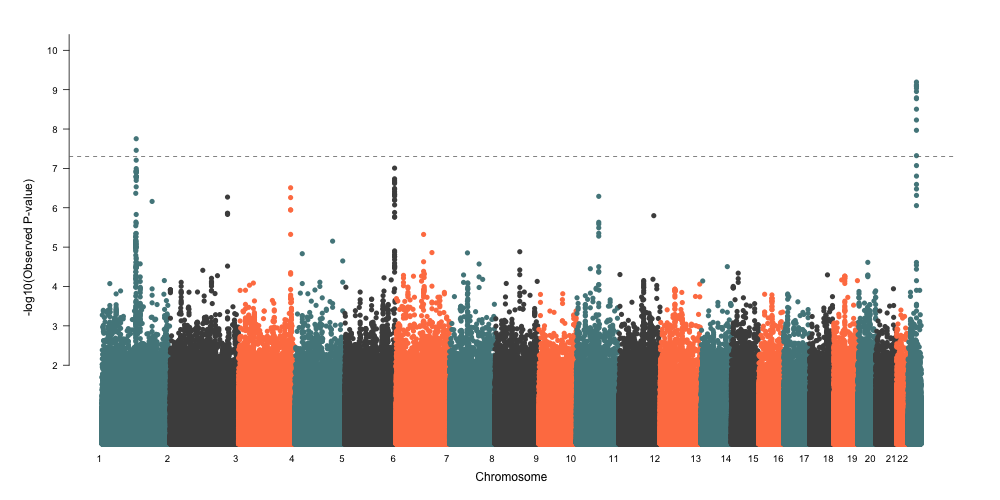


***Supplementary Figure 3: Manhattan Plot of Genome-wide Association Results by Chromosome.*** *Significance is shown along the y-axis with the –log_10_ transformation of the GWAS P-values. Each dot signifies one SNP. The grey line indicates genome-wide significance at 5x10^-8^. SNPs are organized by chromosome (different colors) and position along the y-axis.*


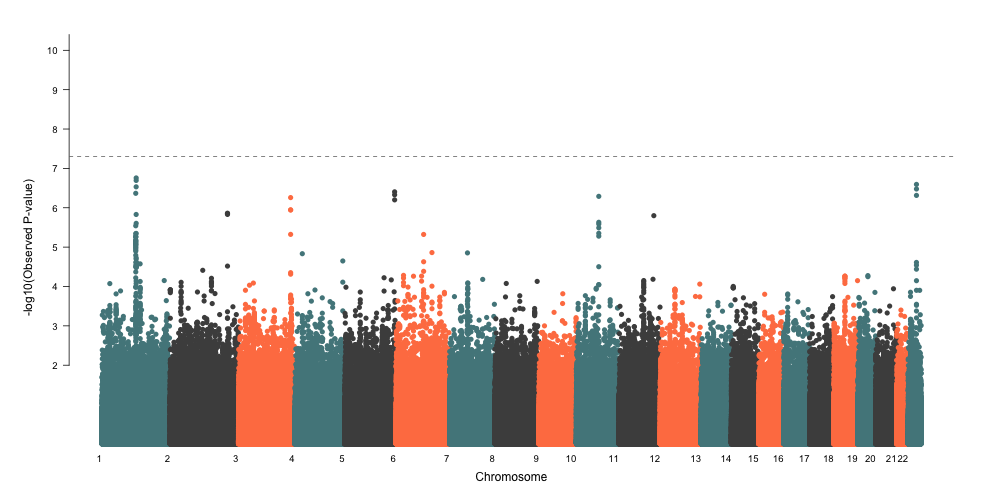
***Figure S4: Manhattan Plot of SNPs with an effect size below 1.25 by chromosome.*** *Significance is shown along the y-axis with the –log10 transformation of the GWAS P-values. The grey line indicates genome-wide significance at 5x10-8. SNPs are organized by chromosome (different colors) and position along the y-axis.*


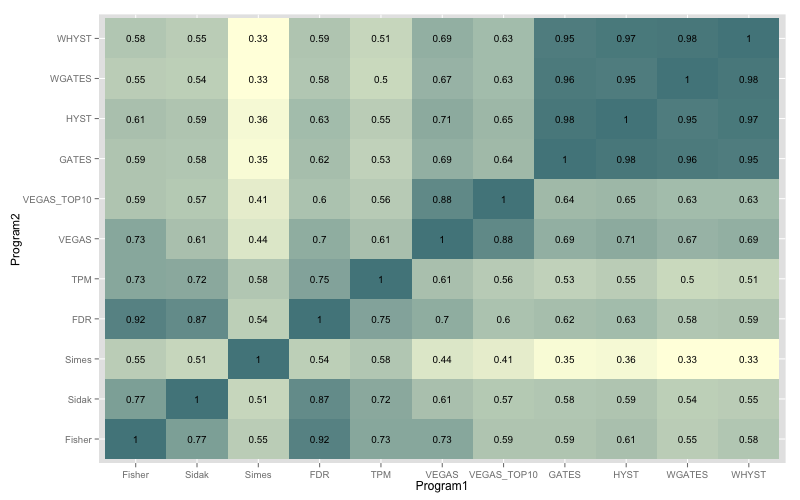


***Figure S5: Genome-wide Correlation of P-values for Gene-Level Methods***


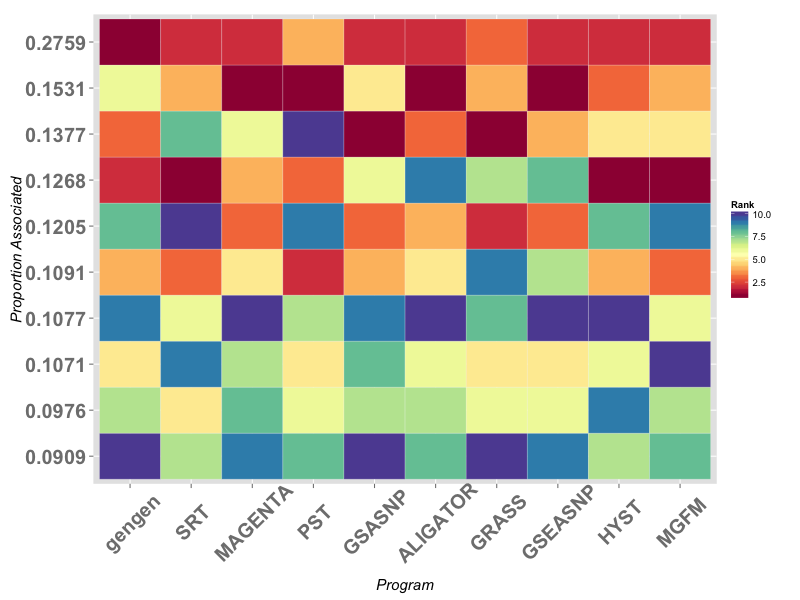


***Figure S6: Ranking of Associations by Programs and Proportion of Genes Associated with a SNP with P<0.01 for the 10 Larger Pathways.***

***
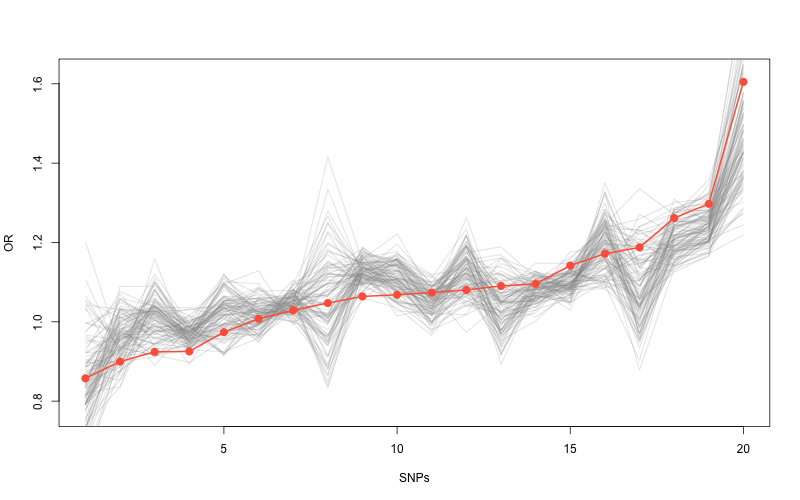
***

***Figure S7: Replicate simulations of phenotype and comparison to simulation used in analysis for a subset of tagSNPs. Simulated effect sizes are shown in grey with the y-axis representing the resulting effect sizes. The red dots show the OR from the original simulation.***

***
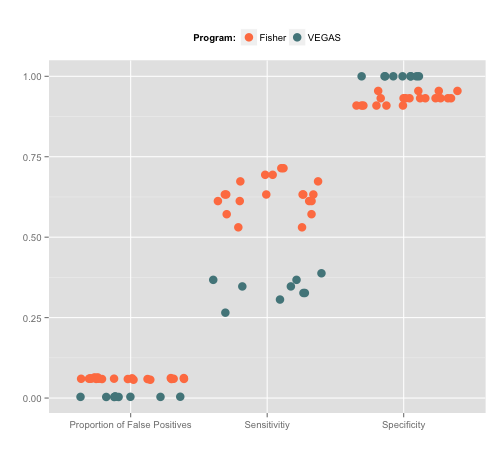
***

***Figure S8: Stability of simulations for gene-level programs VEGAS (top 10%) and Fisher’s Combination Test.***

***
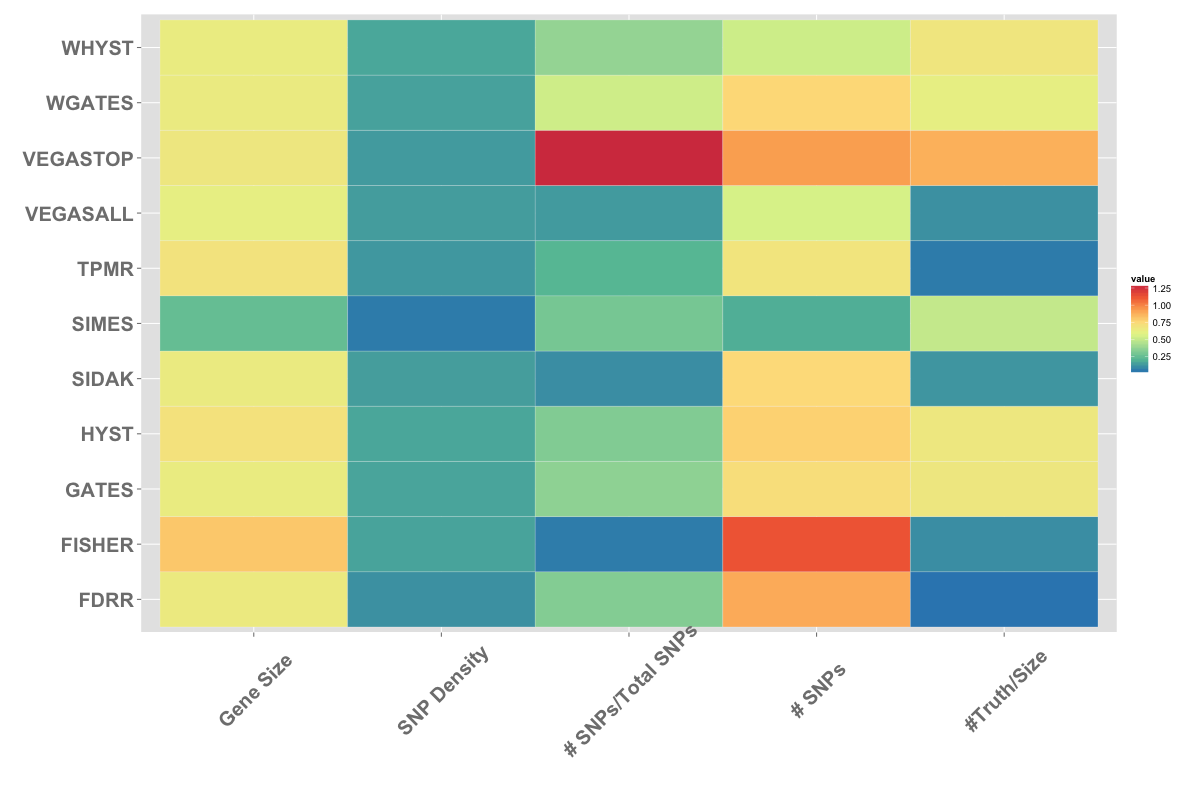
***

***Figure S9: -Log10 P-values of association between Variables and Agreement with Simulation for Gene-Level Programs.***  *Variables include Gene Size (kb), SNP Density (# SNPs/kb), Proportion Causal SNPs to Total SNPs, Total Number of SNPs, and the Ratio of Causal SNPs to kb.*

## Supplementary Methods

### Gene-Level Methods

1. **Fisher’s Combination Test:** Fisher’s combination test (FCT) takes the natural log of the SNP P-values, summing across all SNPs in the gene, and then multiplies by -2. The resulting chi-squared test statistic’s degrees of freedom is determined by the number of SNPs in the gene.[1]
2. **Sidak’s Combination Test**: Sidak’s Combination Test, also called Sidak’s Correction, takes the minimum SNP from the gene and corrects for the number of SNPs.[1]
3. **Simes’ Test:** SNPs are ordered from the most to least significant, multiplied by the total number of SNPs, and divided by their rank. The minimum transformed P-value is then used as the gene-level P-value.[1]
4. **False Discovery Rate (FDR):** The SNP P-values are ordered from most to least significant and are corrected for the False Discovery Rate. The minimum False Discovery Rate is then used as the gene-level output.[1]
5. **Truncated Product Method (TPM):** The truncated product method (TPM) is an adaptation of the Fisher’s Combination Test in which only P-values below a certain threshold are incorporated into the analysis. Values of 0.1, 0.2, and 0.5 were used on this dataset, with the highest sensitivity and lowest proportion of false positives occurring with a truncation value of 0.1. These are the results presented in this manuscript. [2]
6. **GATES/Weighted GATES:** SNP P-values are assessed for correlations and independent representative SNPS are selected for each gene. The representative SNPs are then corrected using the Simes’ procedure. The Weighted GATES methods incorporates weights for the SNPs depending on their functional relevance (intron, exon, nonsynonymous, etc).[3]
7. **HYST/Weighted HYST:** HYST is part of the GATES package in which a modified hypergeometric test is used to determine a gene-level test statistic for enrichment. The weighted HYST procedure weights SNPs based on their functional relevance.[4]
8. **VEGAS (All/Top 10%):** VEGAS directly estimates the correlation structure of the genes by using a Cholesky decomposition. Permutations are conducted to determine an empirical P-value. All SNPs can be used within the gene, or just the top 10% of associated SNPs within each gene.[5]

### Pathway-Level Methods

##### Competitive Methods

1. **ALIGATOR**[6] : ALIGATOR is a method that looks for the enrichment of significant genes within Gene Ontology gene sets. The input is SNP p-values. ALIGATOR then filters by a pre-set P-value threshold (p < 0.05). Any gene that has at least one SNP below this P-value threshold is annotated as being “significant”. Simulations are then conducted in which SNPs are randomly drawn from the GWAS and if they are in a gene that gene is added to the simulated gene list. This is repeated until the gene list is the same length as the original study’s significant gene list. This process is repeated to form 5,000 null gene lists. An empirical p-value is then calculated from the distribution of these gene lists in GO pathways. Because of this simulation procedure, this method is categorized as competitive. Multiple comparisons issues are controlled using a bootstrap procedure. This method is dependent upon all genes within a GO set having comparable linkage disequilibrium patterns. When a gene set has higher levels of linkage disequilibrium, the estimate tends to be overconservative. A total of 1,000 permutations were used in this analysis.
2. **GenGen**[7]: GenGen is the oldest method available, using a modified GSEA which was originally developed for gene expression analyses. The most significant SNP is assigned as the gene’s overall P-value. Genes are then sorted by their significance from smallest to largest p-value. Using these rankings, a Weighted Komogorov-Smirnov-like running sum Enrichment Score (ES) is calculated to see the overrepresentation of highly ranked genes within the gene set. Phenotype permutation adjusts for gene size biases. The original ES is the normalized by the permutations’ enrichment scores to form a Normalized Enrichment Score (NES). A False Discovery Rate (FDR) or a Family-Wise Error Rate (FWER) can be used to control for multiple comparisons. This method is also competitive. A thousand permutations are used to calculate the normalized enrichment scores.
3. **GSA-SNP**[8]: GSA-SNP is an updated method adapted from gene expression studies. It uses the –log transformed SNP p-values as an input and the *k*^th^ most significant SNP is selected as the gene-level P-value (default *k*=2). This is to minimize the effect of spurious associations for the top SNP in the summarization of gene-level statistics. Three different methods are then offered within the package: (1) Z-score, (2) Restandardized-GSA, and (3) GSEA. The Z-score compares the average gene score within the gene set to an overall distribution. Both the Restandardized-GSA and GSEA use permutations to assess significance with pooled set scores. GSA-SNP is available as a graphical user interface (GUI).
4. **GSEA-SNP**[8] : A direct adaptation of the original GSEA algorithm[9], GSEA-SNP uses the raw genotypes as an input. Three inheritance models (recessive, dominant, and additive) are used and the most significant test statistic is calculated per SNP. These test statistics are then ranked genome-wide. Using a running sum statistic, an enrichment score is calculated to determine if a gene set’s SNPs are overrepresented at the top of the genome-wide SNP list. This ES is tnormalized by the gene size to establish a Normalized Enrichment Score (NES). A false discovery rate is calculated to control for false positives. For this project, GSEA-SNP was conducted as part of the SNPath package within R (<http://linchen.fhcrc.org/grass.html>).
5. **MAGENTA**[11]: MAGENTA requires SNP P-values as input, mapping SNPs to genes and using the most significant SNP P-value within that gene as the raw gene-level P-value. Gene p-values can be adjusted for multiple confounders, such as gene size, using regression and permutations. The adjusted gene-level P-values are ranked and “significant” genes are selected using a static cut-off, such as the 95^th^ percentile. Gene sets are checked against this list of significant genes for over-enrichment, similar to a standard GSEA analysis. The rank can also be decreased if a polygenic model is hypothesized (i.e.75^th^ percentile and up).
6. **Modified Generalized Fisher’s Method (MGFM)** [12]: MGFM is a generalization of Fisher’s Combination Test through a modification of Lancaster’s test statistic. Using SNP P-values as input, it takes into account correlation structure of SNPs due to linkage disequilibrium. In addition, there are four weight functions available: (1) adjust for gene size, (2) upweight common SNPs, (3) upweight rare SNPs, and (4) standard Fisher’s Method with identical weights for each SNP. The results presented in this manuscript used the first weighting function, which removed bias due to larger gene sizes. The other weighting functions were not presented due to the absence of rare variants in the dataset, and the higher type I error found using the traditional Fisher’s Combination Test.
7. **SNP Ratio Test** (SRT)[13]: The SNP Ratio Test requires SNP P-values as input, as well as the SNP P-values from permutations calculated using Plink. Using a p-value threshold determined by the user, the ratio of significant SNPs to the number of all SNPs within a pathway is calculated. Gene-level classifications are ignored. Using permutations, an empirical p-value is calculated for the distribution of this ratio. The P-value threshold for SNP significance can be adjusted depending on the hypothesis. For example, a lower P-value threshold (0.01) would assume numerous smaller effects being important in contrast to a few large effects with a more stringent threshold (P=0.001). A total of 1,000 permutations were conducted in this simulation evaluation study.

##### Self-Contained Tests

1. **GRASS** [14]: GRASS requires raw genotypes to directly estimate the genetic architecture of the genes involved in the evaluated gene sets/pathways. Within each gene, a Principal Components Analysis (PCA) is conducted to determine the SNPs that represent the unique linkage disequilibrium patterns. These “nontrivial” SNPs are then fed into a Group Ridge Regression with Lasso penalty to determine the “most representative eigenSNPs” in regards to their association with disease risk. A gene set association is then conducted by summarizing all of the effects from these “most representative eigenSNPs” across an entire gene set. Permutations are used to create a null distribution and calculate a P-value. For this analysis 1,000 permutations were used.
2. **HYST** [4] : HYST is an extension to the gene-level method of GATES. [3] HYST uses the same graphical user interface (GUI) as GATES (KGG2.5). GATES is an extended Simes procedure to assess gene-level associations that directly accounts for linkage disequilibrium patterns by selecting “independent” SNPs. After performing GATES, HYST uses a scaled chi-square test to assess significance on the GATES P-values output., similar to the Fisher’s Combination Test used in gene-level analyses. Prior weights can be incorporated into the blocks, or genes, if appropriate.
3. **Plink Set Test**[15] : The Plink Set Test assesses the joint significance of a set of SNPs, whether they be within a gene, or within a pathway. Using raw genotype data, the linkage disequilibrium patterns are estimated using all SNPs in a region. After single SNP-association testing, only SNPs below a certain P-value threshold are selected. Then, in decreasing order of significance, “independent” SNPs within that set are selected to be representative of the overall genetic variation in that region using the original LD patterns estimated from the raw genotype data. The average statistic within these “independent” SNPs is then used as the original set statistic. Permutation of the phenotype is conducted to determine an empirical p-value for the set.

### References

1. Peng Q, Zhao J, Xue F: **A gene-based method for detecting gene-gene co-association in a case-control association study**. *European Journal of Human Genetics* 2009, **18**:582–587.

2. Zaykin DV, Zhivotovsky LA, Westfall PH, Weir BS: **Truncated product method for combining P-values.** *Genet Epidemiol* 2002, **22**:170–185.

3. Li M-X, Gui H-S, Kwan JSH, Sham PC: **GATES: A Rapid and Powerful Gene-Based Association Test Using Extended Simes Procedure**. *Am J Hum Genet* 2011, **88**:283–293.

4. Li M-X, Kwan JSH, Sham PC: **HYST: A Hybrid Set-Based Test for Genome-wide Association Studies, with Application to Protein-Protein Interaction-Based Association Analysis**. *Am J Hum Genet* 2012, **91**:478–488.

5. Liu JZ, Mcrae AF, Nyholt DR, Medland SE, Wray NR, Brown KM, Investigators A, Hayward NK, Montgomery GW, Visscher PM, Martin NG, Macgregor S: **A Versatile Gene-Based Test for Genome-wide Association Studies**. *Am J Hum Genet* 2010, **87**:139–145.

6. Holmans P, Green EK, Pahwa JS, Ferreira MAR, Purcell SM, Sklar P, Owen MJ, Donovan MCO, Craddock N, Consortium9 TWTC-C: **Gene Ontology Analysis of GWA Study Data Sets Provides Insights into the Biology of Bipolar Disorder**. *The American Journal of Human Genetics* 2009, **85**:13–24.

7. Wang K, Li M, Bucan M: **Pathway-Based Approaches for Analysis of Genomewide Association Studies**. *The American Journal of Human Genetics* 2007, **81**:1278–1283.

8. Nam D, Kim J, Kim SY, Kim S: **GSA-SNP: a general approach for gene set analysis of polymorphisms**. *Nucleic Acids Research* 2010, **38**(Web Server):W749–W754.

9. Holden M, Deng S, Wojnowski L, Kulle B: **GSEA-SNP: applying gene set enrichment analysis to SNP data from genome-wide association studies**. *Bioinformatics* 2008, **24**:2784–2785.

10. Subramanian A, Tamayo P, Mootha VK, Mukherjee S, Ebert BL, Gillette MA, Paulovich A, Pomeroy SL, Golub TR, Lander ES, Mesirov JP: **Gene set enrichment analysis: A knowledge-basedapproach for interpreting genome-wideexpression profiles**. *Proceedings of the National Academy of Sciences* 2005, **102**:15545–15550.

11. Segrè AV, DIAGRAM Consortium, MAGIC investigators, Groop L, Mootha VK, Daly MJ, Altshuler D: **Common Inherited Variation in Mitochondrial Genes Is Not Enriched for Associations with Type 2 Diabetes or Related Glycemic Traits**. *PLoS Genetics* 2010, **6**:e1001058.

12. Dai H: **A modified generalized Fisher method for combining probabilities from dependent tests**. 2014:1–10.

13. O'Dushlaine C, Kenny E, Heron EA, Segurado R, Gill M, Morris DW, Corvin A: **The SNP ratio test: pathway analysis of genome-wide association datasets**. *Bioinformatics* 2009, **25**:2762–2763.

14. Chen LS, Hutter CM, Potter JD, Liu Y, Prentice RL, Peters U, Hsu L: **Insights into Colon Cancer Etiology via a Regularized Approach to Gene Set Analysis of GWAS Data**. *The American Journal of Human Genetics* 2010, **86**:860–871.

15. Purcell S, Neale B, Todd-Brown K, Thomas L, Ferreira MAR, Bender D, Maller J, Sklar P, de Bakker PIW, Daly MJ, Sham PC: **PLINK: A Tool Set for Whole-Genome Association and Population-Based Linkage Analyses**. *The American Journal of Human Genetics* 2007, **81**:559–575.
